# Effect of *in ovo*-fed amino acids on muscle and liver metabolome of broiler chickens at 24 h post-hatch

**DOI:** 10.3389/fphys.2025.1542426

**Published:** 2025-04-25

**Authors:** Moustafa Yehia, Angel Rene Alfonso-Avila, Jean-Michel Allard Prus, Véronique Ouellet, Nabeel Alnahhas

**Affiliations:** ^1^ Department of Animal Science, Faculty of Agricultural and Food Sciences, Université Laval, Quebec City, QC, Canada; ^2^ Deschambault Research Center in Animal Science (CRSAD), Deschambault, QC, Canada; ^3^ Scott Hatchery, Scott, QC, Canada; ^4^ Swine and Poultry Infectious Diseases Research Center, Université de Montréal, Saint-Hyacinthe, QC, Canada

**Keywords:** broiler, *in ovo* feeding, amino acids, amino acid metabolism, thermoregulation

## Abstract

*In ovo* administration of amino acids has been shown to alleviate the adverse effects of heat stress on broiler chickens during the finisher phase. However, their specific influence on thermogenic organs in the early post-hatch period is not fully understood. Therefore, the aim of the present study was to explore and investigate the effects of *in ovo*-fed amino acids on amino acid metabolism in the liver and muscle of one-day-old broiler chicks. To achieve this, breast muscle and liver samples were taken from six randomly selected chicks per experimental group and subjected to a targeted metabolomic analysis. The experimental groups included a control group injected with 52 µL of sterile diluent/egg (CTRL), a group injected with 3.0 mg of L-Met + 2.0 mg of L-Cys/egg (T1), and a group injected with 0.4 mg of L-Leu + 1.6 mg of L-Met + 1.6 mg of L-Cys/egg (T2). The Sparse Partial Least Square – Discriminant Analysis (sPLS-DA) showed that T1 and T2 had very similar metabolomic profiles. Consequently, data from T1 and T2 were merged into a single group (Injected) for statistical analysis. Compared to CTRL, multiple pathways were significantly enriched in the muscle and liver of the Injected group. These enriched pathways included those involved in the metabolism of cysteine and methionine (FDR = 0.01), glutathione (FDR < 0.001), histidine (FDR = 0.01), taurine (FDR = 0.01), glycine, serine, and threonine (FDR = 0.01) as well as the pathway of arginine biosynthesis (FDR = 0.03). Moreover, only four muscle metabolites: homocysteine (r = −0.63, P = 0.03), S-Adenosyl-homocysteine (r = −0.62, P = 0.03), phosphocholine (r = 0.50, P = 0.01), and betaine (r = 0.52, P = 0.004), as well as four liver metabolites: phenyl pyruvic acid (r = 0.55, P = 0.02), dimethylglycine (r = 0.55, P = 0.03), phenylalanine (r = 0.50, P = 0.02), and alpha-aminobutyric acid (r = −0.53, P = 0.02) were significantly correlated with the rectal temperature of sampled chicks, suggesting a role of these metabolites in thermoregulation. In conclusion, the *in ovo* feeding of amino acids on embryonic day 18 was associated with the enrichment of pathways directly or indirectly involved in the response of the antioxidant defense system to oxidative stress in the liver and muscle tissues.

## 1 Introduction

Selective breeding efforts to improve broiler growth rate, meat yield, and feed efficiency are associated with elevated metabolic rate and heat production (Nascimento et al., 2017). Since chickens inherently lack sweat glands and are insulated by their feathers, such selection-induced increases in heat production have exacerbated broiler susceptibility to heat stress (Tabler et al., 2020; Malila et al., 2021). Furthermore, rising surface temperatures caused by global warming have designated heat stress as an important challenge for the poultry industry. Consequently, the anticipated economic repercussions of heat stress on poultry production are expected to worsen in the coming years (Kumar et al., 2021).

The importance of addressing this challenge in the poultry industry stems from the deleterious consequences of exposure to high ambient temperatures on broiler zootechnical performance. Research has shown that exposure to temperatures above the upper critical limit of broiler thermoneutral zone (i.e., heat stress) impairs growth performance in modern broiler strains by reducing weight gain through decreased feed intake and resource reallocation to inflammatory and oxidative processes (Goo et al., 2019; Awad et al., 2020). Such exposure also accelerates muscle post-mortem glycolysis, leading to a reduction in muscle post-mortem pH. These conditions induce oxidative damage in the pectoral muscles, ultimately resulting in a deterioration in meat quality (Zaboli et al., 2019). Moreover, heat stress reduces the capacity of the immune system to produce an effective immune response when challenged (Monson et al., 2018; Hirakawa et al., 2020), which renders broilers more susceptible to disease and amplifies economic losses due to high mortality rates.

*In ovo* feeding of branched-chain and sulfur amino acids has been previously shown to improve energy metabolism and tolerance to higher temperatures (Han et al., 2017; Kop-Bozbay and Ocak, 2019). In a previous study (Yehia et al., 2024), we demonstrated that *in ovo* feeding of a combination of methionine and cysteine (3.0 mg of L-Met + 2.0 mg of L-Cys/egg) and a combination of leucine, methionine, and cysteine (0.4 mg of L-Leu +1.6 mg of L-Met +1.6 mg of L-Cys/egg) were associated with significantly lower facial temperatures compared to the control group (sterile diluent only), under heat stress conditions (34°C, 55%–60% relative humidity, 10 h/d from d 29 to d 34). These two treatments were also associated with lower levels of lipid peroxidation in both the serum and liver. Hence, we concluded that *in ovo* feeding with branched-chain amino acids (i.e., L-Leu) and sulfur-containing amino acids (i.e., L-Met and L-Cys) afforded birds improved tolerance to heat stress in the finisher phase (d 29 – d 35).

The mechanisms underlying the effects of *in ovo* feeding of amino acids on broiler heat tolerance later in life are not fully understood. The present study is the first in a series of studies aiming to identify and elucidate these mechanisms. It aims to unravel the underlying changes in amino acids metabolism induced by the *in ovo* feeding of the above-mentioned treatments from our previous study in the breast muscle and the liver at 24 h post-hatch under thermoneutral conditions. Our reasoning here is that if these treatments induce changes during the late phase of embryogenesis leading to an improved heat tolerance in the finisher phase, these changes would be more evident to unravel early post-hatch. We hypothesize that the *in ovo* feeding of our amino acid treatments on embryonic day (ED) 18 will significantly alter pathways involved in the antioxidant response in the muscle and liver at 24 h post-hatch as compared to control.

## 2 Materials and methods

### 2.1 Experimental treatments

A total of 2,700 fertile eggs were obtained from a flock of Ross 308 broiler breeders (60 weeks of age) and were incubated under optimal conditions from ED 1 to ED 18 in a multistage incubator without any intervention or manipulation (Scott Hatchery, Scott, Quebec, Canada). On ED 18, three solutions were prepared, and each one was injected into 900 eggs chosen at random. The solutions included a control group (CTRL, 52 µL of sterile diluent/egg), T1 (3.0 mg of L-Met +2.0 mg of L-Cys/52 µL of sterile diluent/egg), and T2 (0.40 mg of L-Leu +1.60 mg of L-Met +1.60 mg of L-Cys/52 µL of sterile diluent/egg). The sterile diluent used to prepare the treatments was the solution used at the hatchery to prepare vaccine solutions destined for *in ovo* vaccination (Boehringer Ingelheim Animal Health, USA). All amino acids were purchased from Sigma-Aldrich (L8912, M5308 and C7352 for L-Leu, L-Met and L-Cys, respectively). The selection of the T1 and T2 treatments was based on their significant impact on broiler facial temperature and lipid peroxidation seen in our previous study (Yehia et al., 2024). The *in ovo* treatments were injected into the eggs in the amniotic fluid through the air chamber using an *in ovo* injection robot (Embrex® Inovoject® JW84 System, Zoetis, Canada). On the day of hatch, hatched chicks were counted, wing-sexed, vaccinated against infectious bronchitis by spray (Massachusetts-type strain), and placed in pre-identified transportation boxes according to treatment group replicates. Next, sexed chicks were transported over 90 km to the Deschambault Research Center in Animal Science (Deschambault, Quebec, Canada) and treatment group replicates were placed in a broiler house according to a complete randomized block design consisting of 60 pens of 45 chicks per pen. Male and female chicks were placed in separate pens equipped with bell drinkers and manual feeders. Standard environmental conditions (temperature and humidity) and lighting programs meeting the requirement of Ross 308 were applied and chicks were fed *ad libitum* with a standard starter diet.

### 2.2 Sampling

For the purposes of the present study, a subset of 6 chicks per group were randomly chosen at 24 h post-hatch. First, the rectal temperature was measured using a digital thermometer (Ear digital thermometer model). Next, chicks were weighed, euthanized by cervical dislocation and a sample (∼100 mg) of the Pectoralis major muscle (latero-cranial section) and of the liver were taken, placed on dry ice, and transported back to the laboratory where they were stored at −80°C until analysis (n = 18 muscle and 18 liver samples).

### 2.3 Metabolomic analyses

Samples were then shipped on dry ice to the Metabolomic Platform of Genome British Colombia (University of Victoria, BC, Canada) for targeted analysis of amino acid metabolism. First, the tissue samples were precisely weighed into 2-mL Eppendorf homogenization tubes. Then, water (what kind of water) was added to each sample at 3 μL per mg of raw tissue. The samples were then homogenized on a MM 400 mixer mill at 30 Hz for 30 min with the aid of two 4-mm metal balls. After short spin-down on a microcentrifuge, acetonitrile was added to each sample at 7 μL per mg of raw tissue. Then, the samples were homogenized again for 3 min followed by ultrasonication for 3 min in an ice-water bath. Finally, the samples were centrifuged at 21,000 g and 10°C for 10 min. The clear supernatants were collected and used for the following metabolomics assays.

#### 2.3.1 Quantitation of amino acids/amines

A stock solution of the targeted metabolites was prepared with their standard substances in 70% acetonitrile. This solution was serially diluted with the same solvent to have 9-point calibration solutions, with the concentrations for each metabolite in a range of 0.00005–50 μM. The clear supernatant of each sample was diluted 20-fold with 70% acetonitrile. 20 μL aliquots of the clear diluted and undiluted supernatants of each sample and 20 μL of each of the calibration solutions were mixed with 20 μL of an internal standard solution of 40 isotope-labeled amino acids/amines, 40 μL of a 10-mM dansyl chloride solution, and 40 μL of a borate buffer at pH 9. The mixtures were incubated at 40°C for 40 min. After incubation, 5 μL of each solution was injected into a 15-cm long C18 UPLC column to run LC-MRM/MS on an Agilent 1290 UHPLC instrument coupled to an Agilent 6495C QQQ mass spectrometer, which was operated with positive-ion detection. Water containing 0.1% formic acid and acetonitrile-isopropanol (1:1) containing 0.1% formic acid were used as the mobile phase for binary-solvent gradient elution under optimized conditions of LC separation and MRM/MS detection.

#### 2.3.2 Quantitation of other metabolites

A stock solution of the targeted metabolites was prepared with their standard substances in 70% acetonitrile. This solution was serially diluted with the same solvent to have 9-point calibration solutions with the concentration range of 0.00001–10 μM for each metabolite. 20 μL of the clear supernatant of each sample or 20 μL of each calibration solution was mixed with 180 μL of an internal standard solution of 12 isotope-labeled metabolites. 10 μL aliquots of the resultant solutions were injected into a 10-cm long C18 UPLC column to run LC-MRM/MS on an Agilent 1290 UHPLC instrument coupled to an Agilent 6495C QQQ mass spectrometer and operated with positive-ion detection. A heptafluorobutyric acid solution and acetonitrile were used as the mobile phase for binary-solvent gradient elution under optimized conditions of LC separation and MRM/MS detection.

#### 2.3.3 Quantitation of organic acid type metabolites

A stock solution of the targeted metabolites was prepared with their standard substances in 70% acetonitrile. This solution was serially diluted with the same solvent to have 9-point calibration solutions with the concentration range being 0.00001–10 μM for each metabolite. 20 μL of the clear supernatant of each sample or 20 μL of each calibration solution was mixed with 20 μL of an internal standard solution of 13C3-pyruvic acid, 80 μL of 100-mM 3-nitrophenylhydrazine solution and 80 μL of 100-mM EDC-4% pyridine solution. The mixtures were incubated at 50°C for 40 min 10 μL aliquots of the resultant solutions were injected into a 10-cm long C18 UPLC column to run LC-MRM/MS on an Agilent 1290 UHPLC instrument coupled to an Agilent 6495B QQQ mass spectrometer, which was operated with negative-ion detection. 0.01% formic acid in water and acetonitrile were used as the mobile phase for binary-solvent gradient elution under optimized conditions of LC separation and MRM/MS detection.

For the above assays, linear regression calibration curves of individual metabolites were constructed with the data acquired from the calibration solutions. Concentrations of detected metabolites were calculated from the calibration curves with the data acquired from the sample solutions.

### 2.4 Data preprocessing and analysis

First, raw concentrations of analyzed metabolites were median-centered and log-transformed using the *Normalization* function of MetaboAnalyst (V4.0) R package (Pang et al., 2024b) to reduce the influence of variation in the concentration of different metabolites on the subsequent analysis. Next, a Sparse Partial Least Square–Discriminant Analysis (sPLS-DA) as implemented in the R package *mixOmics* (Rohart et al., 2017) was fitted to the normalized data using the function *splsda* with the number of components set to two. Then, we used a variable selection procedure based on a cross-validation scheme as implemented in the function *tune. splsda* from the same R package to determine the final set of metabolites that maximized the discrimination (i.e., the distance) between the experimental groups for muscle and liver samples. To achieve this, a 5-fold cross validation scheme repeated 100 times was used. We then used the function *plotLoadings* from the *mixOmics* package to extract the contribution (i.e., loading weights) of the model-selected metabolites to determine the experimental groups in which each selected metabolite was the most abundant. To understand the biological significance of the metabolites selected by the model, we conducted a pathway enrichment analysis to identify the KEGG pathways in which these metabolites were enriched using a hypergeometric test as implemented in the web version of MetaboAnalyst (Pang et al., 2024a). Finally, a correlation analysis was conducted between muscle and liver metabolites, on one hand, and the rectal temperature of sampled chicks, on the other hand, using the *rcorr* function from the *Hmisc* package in R, to explore associations with metabolites that may relate to this temperature.

An analysis of variance was conducted for hatchability (n = 6 hatching trays/group), body weight (n = 20 chicks/group) and rectal temperature (n = 20 chicks/group) at 24 h post-hatch using the *aov* function of R. The model included the treatment, sex and their interaction (the sex and sex-by-treatment interaction effects were only fitted for body weight and rectal temperature) as fixed effects. Means of these effects were separated and differences between them were tested for significance using the *emmeans* package of R. The results of this analysis were presented as least squares means and their standard errors. Differences between means were declared significant at *P* < 0.05.

## 3 Results

### 3.1 Hatchability, body weight, and rectal temperature

**FIGURE 1:**
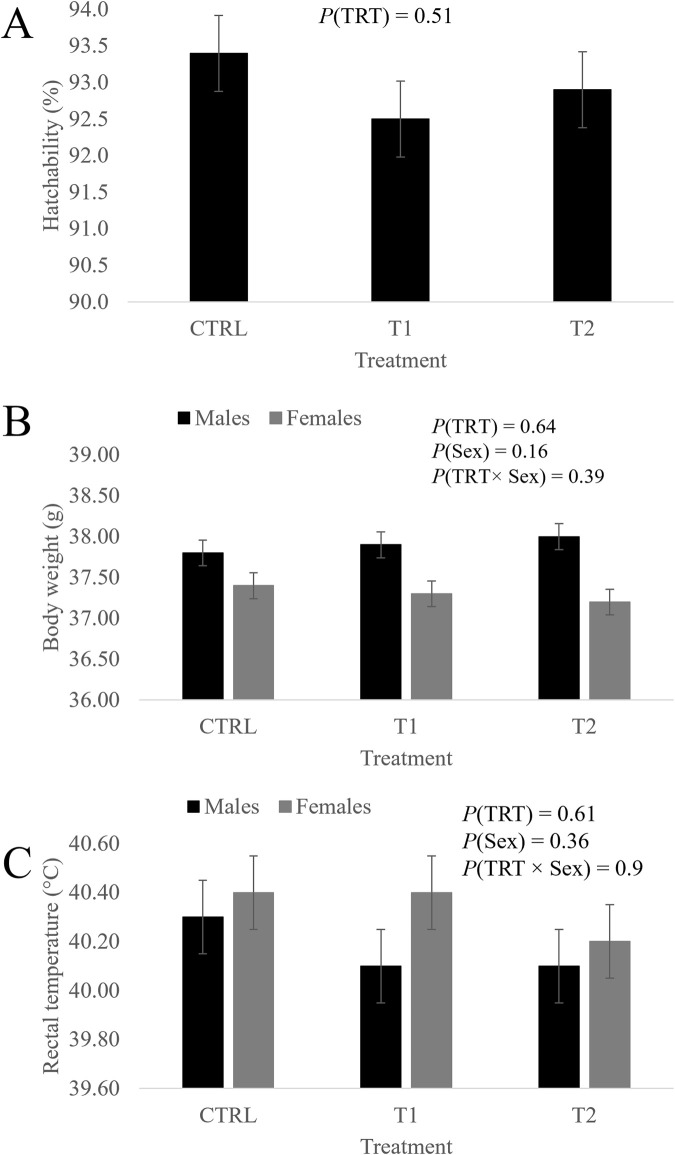
Effect of treatment, sex and their interaction on hatchability **(A)**, body weight **(B)** and rectal temperature **(C)** of day-old chicks. CTRL is the control group (52 µL of sterile diluent/egg), T1 (3.0 mg of L-Met +2.0 mg of L-Cys/52 µL of sterile diluent/egg) and T2 (0.40 mg of L-Leu +1.60 mg of L-Met +1.60 mg of L-Cys/52 µL of sterile diluent/egg) are the *in ovo*-fed groups. Data is presented as the least squares means and their standard errors.

These traits were not influenced by the effects of the treatment, the sex or the sex-by-treatment interaction (Figure 1).

### 3.2 SPLS-DA analysis

**FIGURE 2:**
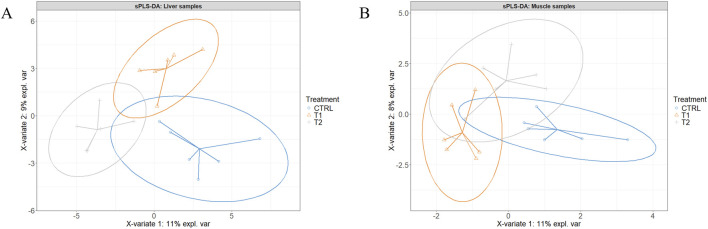
Partial least squares-discriminant analysis of the liver **(A)** and the Pectoralis major muscle **(B)** of the control (CTRL, 52 µL of sterile diluent/egg) and experimental T1 (3.0 mg of L-Met +2.0 mg of L-Cys/52 µL of sterile diluent/egg), and T2 (0.40 mg of L-Leu +1.60 mg of L-Met +1.60 mg of L-Cys/52 µL of sterile diluent/egg) groups.

**FIGURE 3:**
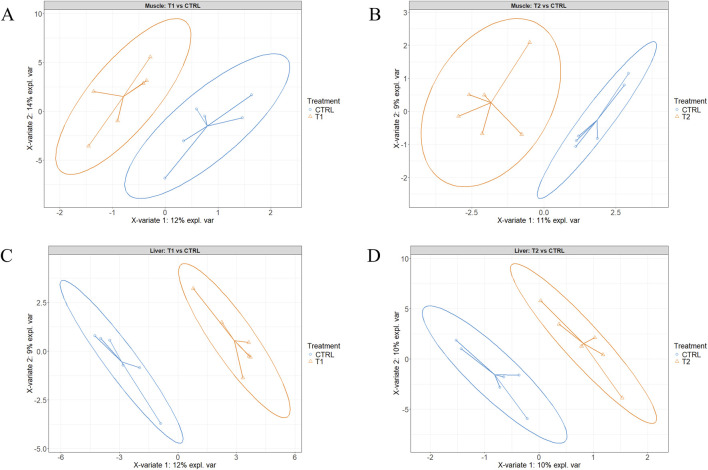
Partial least squares-discriminant analysis of the Pectoralis major muscle **(A, B)** and the liver **(C, D)** tissue samples. **A** and **C** T1 (3.0 mg of L-Met +2.0 mg of L-Cys/52 µL of sterile diluent/egg) vs. the control (52 µL of sterile diluent/egg), **B** and **D** T2 (0.40 mg of L-Leu +1.60 mg of L-Met +1.60 mg of L-Cys/52 µL of sterile diluent/egg) vs. the control group.

**FIGURE 4:**
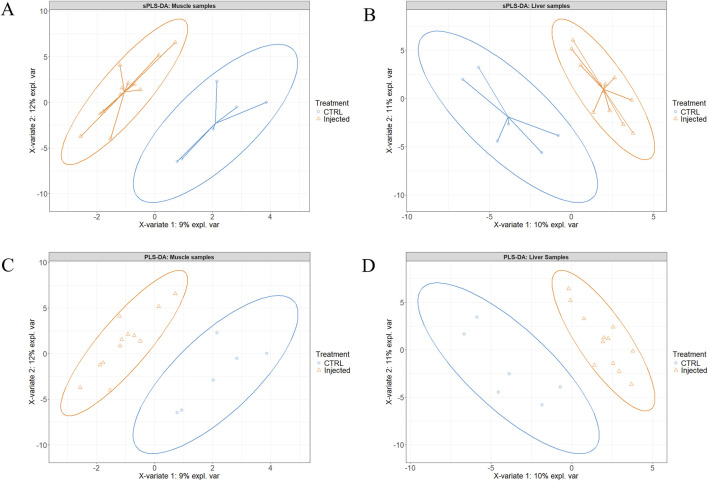
Partial least squares-discriminant analysis of the Pectoralis major muscle **(A, C)** and the liver **(B, D)** tissue samples after merging the experimental T1 (3.0 mg of L-Met +2.0 mg of L-Cys/52 µL of sterile diluent/egg) and T2 (0.40 mg of L-Leu +1.60 mg of L-Met +1.60 mg of L-Cys/52 µL of sterile diluent/egg) groups into a single injected group. Panel **A** and **B** are the sample plots, and panel **C** and **D** are the corresponding individual plots.

Figures 2A,B present the sample plots according to the final sPLS-DA model of liver and muscle tissue, respectively. As illustrated in these two figures, the 95% confidence ellipses of the three treatment groups exhibited partial overlapping. However, when the injected groups were individually compared to the CTRL group using the same model, a clear separation between T2 and T3 on one hand, and CTRL on the other was observed (Figures 3A–D). When the two injected groups were merged into a single group, the separation from the CTRL group remained clear in the muscle and liver tissues (Figures 4A–D).

### 3.3 Between-group discriminatory metabolites

**FIGURE 5:**
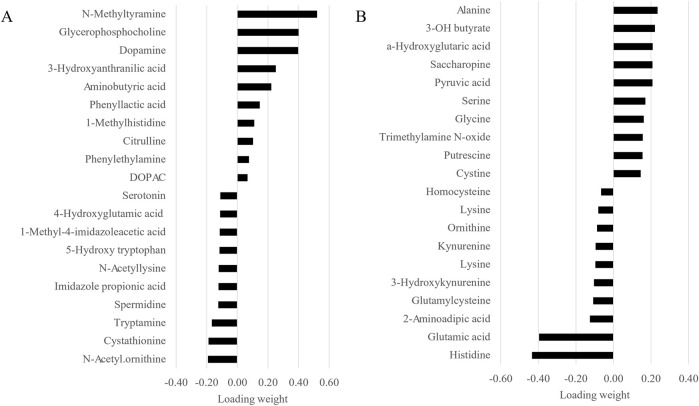
Top 10 muscle metabolites with the highest and lowest loading weights contributing to the control **(A)** and to the injected **(B)** groups.

**FIGURE 6:**
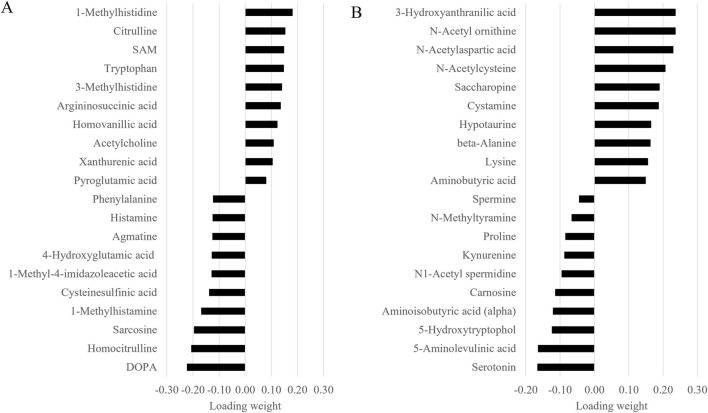
Top 10 liver metabolites with the highest and lowest loading weights contributing to the control **(A)** and to the injected **(B)** groups.

Given the similarity of the metabolomic profiles between T1 and T2 (Figures 2, 4) and the clear separation between these two groups and the CTRL group (Figure 3), we merged the two experimental groups as a single group and then identified the metabolites contributing to the CTRL group and to this new merged injected group (Figures 4A–D). The final model retained 103 metabolites from the 129 metabolites measured in muscle tissue (Supplementary Table S1). Of the retained muscle metabolites, 37 were more abundant in the CTRL group and 66 were more abundant in the injected group according to their loading weights. The top 10 metabolites with the highest and lowest loading weights (i.e., importance) in the CTRL and injected group of the muscle tissue are illustrated in Figures 5A,B, respectively. Regarding the liver, the number of retained metabolites was of 97 metabolites out of the 131 measured metabolites. Of the retained metabolites, 44 were more abundant in the CTRL group and 53 were more abundant in the injected group according to their loading weights (Supplementary Table S2). The top 10 metabolites with the highest and lowest loading weights in the CTRL and injected group of the liver tissue are illustrated in Figures 6A,B, respectively.

### 3.4 Pathway enrichment analysis

Tables 1, 2 present the results of the pathway enrichment analysis using metabolites contributing to the CTRL and injected group of muscle tissue, respectively. Metabolites associated with the CTRL group were enriched across multiple pathways. However, the FDR-corrected P-values from the enrichment analysis indicated that these enrichments were not statistically significant. On the other hand, multiple pathways of specific interest in relation to the treatments used in the current study were significantly enriched in the injected group including glutathione metabolism, and cysteine and methionine metabolism pathway, while the valine, leucine and isoleucine biosynthesis pathways only tended to be significantly enriched.

**TABLE 1:** Overrepresentation pathway enrichment analysis results of metabolites contributing to the control group of muscle samples^a^.

KEGG pathway	Total	Expected	Hits	P-value	FDR
Tryptophan metabolism	41.00	0.51	4.00	0.00	0.10
Phenylalanine metabolism	8.00	0.10	2.00	0.00	0.16
Glycine; serine and threonine metabolism	33.00	0.41	3.00	0.01	0.18
Arginine and proline metabolism	36.00	0.44	3.00	0.01	0.18
Histidine metabolism	16.00	0.20	2.00	0.02	0.25
Beta-Alanine metabolism	21.00	0.26	2.00	0.03	0.35
Glutathione metabolism	28.00	0.35	2.00	0.05	0.49
Phenylalanine; tyrosine and tryptophan biosynthesis	4.00	0.05	1.00	0.05	0.49
Tyrosine metabolism	42.00	0.52	2.00	0.09	0.83
Arginine biosynthesis	14.00	0.17	1.00	0.16	1.00
Ether lipid metabolism	20.00	0.25	1.00	0.22	1.00
Cysteine and methionine metabolism	33.00	0.41	1.00	0.34	1.00
Glycerophospholipid metabolism	36.00	0.44	1.00	0.36	1.00

^a^ Total: Total number of metabolites in the pathway, Expected: Expected number of metabolites in the pathway, Hits: The number of metabolites found in the input list, P-value: Raw P-value of the hypergeometric test, FDR: P-value corrected for the multiplicity of tests.

**TABLE 2:** Overrepresentation pathway enrichment analysis results of metabolites contributing to the *in ovo*-injected group of muscle samples^a^.

KEGG pathway	Total	Expected	Hits	P-value	FDR
Glycine; serine and threonine metabolism	33.00	0.77	8.00	0.00	0.00
Arginine and proline metabolism	36.00	0.84	7.00	0.00	0.00
Glutathione metabolism	28.00	0.66	6.00	0.00	0.00
Arginine biosynthesis	14.00	0.33	4.00	0.00	0.00
Alanine; aspartate and glutamate metabolism	28.00	0.66	5.00	0.00	0.01
Histidine metabolism	16.00	0.37	4.00	0.00	0.01
Glyoxylate and dicarboxylate metabolism	31.00	0.73	5.00	0.00	0.01
Taurine and hypotaurine metabolism	8.00	0.19	3.00	0.00	0.01
Cysteine and methionine metabolism	33.00	0.77	5.00	0.00	0.01
Nitrogen metabolism	6.00	0.14	2.00	0.01	0.06
Valine; leucine and isoleucine biosynthesis	8.00	0.19	2.00	0.01	0.10
Tyrosine metabolism	42.00	0.98	4.00	0.02	0.10
Porphyrin metabolism	31.00	0.73	3.00	0.03	0.21
Butanoate metabolism	15.00	0.35	2.00	0.05	0.26
beta-Alanine metabolism	21.00	0.49	2.00	0.08	0.45
Phenylalanine; tyrosine and tryptophan biosynthesis	4.00	0.09	1.00	0.09	0.45
Lipoic acid metabolism	28.00	0.66	2.00	0.14	0.65
Lysine degradation	30.00	0.70	2.00	0.15	0.69
Phenylalanine metabolism	8.00	0.19	1.00	0.17	0.73
Glycerophospholipid metabolism	36.00	0.84	2.00	0.21	0.81
Biotin metabolism	10.00	0.23	1.00	0.21	0.81
Valine; leucine and isoleucine degradation	39.00	0.91	2.00	0.23	0.84
Tryptophan metabolism	41.00	0.96	2.00	0.25	0.87
Primary bile acid biosynthesis	46.00	1.08	2.00	0.29	0.96
D-Amino acid metabolism	15.00	0.35	1.00	0.30	0.96

^a^ Total: Total number of metabolites in the pathway, Expected: Expected number of metabolites in the pathway, Hits: The number of metabolites found in the input list, P-value: Raw P-value of the hypergeometric test, FDR: P-value corrected for the multiplicity of tests.

Tables 3, 4 summarize the results of the pathway enrichment analysis in the liver tissue samples using metabolites contributing to the CTRL and injected group, respectively. According to the FDR-corrected P-values, only the histidine metabolism and glycine, serine and threonine metabolism pathways were significantly enriched in the CTRL group. In the injected group, pathways including glutathione metabolism and histidine metabolism were among the most enriched pathways.

**TABLE 3:** Overrepresentation pathway enrichment analysis results of metabolites contributing to the control group of liver samples^a^.

KEGG pathway	Total	Expected	Hits	P-value	FDR
Histidine metabolism	16.00	0.31	4.00	0.00	0.01
Glycine; serine and threonine metabolism	33.00	0.64	5.00	0.00	0.01
Arginine biosynthesis	14.00	0.27	3.00	0.00	0.06
Arginine and proline metabolism	36.00	0.70	4.00	0.00	0.07
Glycerophospholipid metabolism	36.00	0.70	4.00	0.00	0.07
Phenylalanine metabolism	8.00	0.16	2.00	0.01	0.13
Tyrosine metabolism	42.00	0.82	3.00	0.05	0.53
Phenylalanine; tyrosine and tryptophan biosynthesis	4.00	0.08	1.00	0.08	0.76
Alanine; aspartate and glutamate metabolism	28.00	0.55	2.00	0.10	0.89
Nitrogen metabolism	6.00	0.12	1.00	0.11	0.89
Cysteine and methionine metabolism	33.00	0.64	2.00	0.13	0.97
Taurine and hypotaurine metabolism	8.00	0.16	1.00	0.15	0.97

^a^ Total: Total number of metabolites in the pathway, Expected: Expected number of metabolites in the pathway, Hits: The number of metabolites found in the input list, P-value: Raw P-value of the hypergeometric test, FDR: P-value corrected for the multiplicity of tests.

**TABLE 4:** Overrepresentation pathway enrichment analysis results of metabolites contributing to the *in ovo*-injected group of liver samples^a^.

KEGG pathway	Total	Expected	Hits	P-value	FDR
Valine; leucine and isoleucine biosynthesis	8.00	0.17	4.00	0.00	0.00
Glutathione metabolism	28.00	0.60	6.00	0.00	0.00
Arginine and proline metabolism	36.00	0.77	6.00	0.00	0.00
Histidine metabolism	16.00	0.34	4.00	0.00	0.01
Taurine and hypotaurine metabolism	8.00	0.17	3.00	0.00	0.01
Glycine; serine and threonine metabolism	33.00	0.71	5.00	0.00	0.01
beta-Alanine metabolism	21.00	0.45	4.00	0.00	0.01
Arginine biosynthesis	14.00	0.30	3.00	0.00	0.03
Pantothenate and CoA biosynthesis	20.00	0.43	3.00	0.01	0.07
Tyrosine metabolism	42.00	0.90	4.00	0.01	0.09
Alanine; aspartate and glutamate metabolism	28.00	0.60	3.00	0.02	0.15
Cysteine and methionine metabolism	33.00	0.71	3.00	0.03	0.21
Valine; leucine and isoleucine degradation	39.00	0.84	3.00	0.05	0.30
Tryptophan metabolism	41.00	0.88	3.00	0.06	0.32
Phenylalanine; tyrosine and tryptophan biosynthesis	4.00	0.09	1.00	0.08	0.44
Nitrogen metabolism	6.00	0.13	1.00	0.12	0.56
Lysine degradation	30.00	0.64	2.00	0.13	0.56
Thiamine metabolism	7.00	0.15	1.00	0.14	0.56
Glyoxylate and dicarboxylate metabolism	31.00	0.67	2.00	0.14	0.56
Porphyrin metabolism	31.00	0.67	2.00	0.14	0.56
Phenylalanine metabolism	8.00	0.17	1.00	0.16	0.61
Biotin metabolism	10.00	0.21	1.00	0.20	0.71
D-Amino acid metabolism	15.00	0.32	1.00	0.28	0.93
Butanoate metabolism	15.00	0.32	1.00	0.28	0.93

^a^ Total: Total number of metabolites in the pathway, Expected: Expected number of metabolites in the pathway, Hits: The number of metabolites found in the input list, P-value: Raw P-value of the hypergeometric test, FDR: P-value corrected for the multiplicity of tests.

### 3.5 Temperature correlated metabolites

**FIGURE 7:**
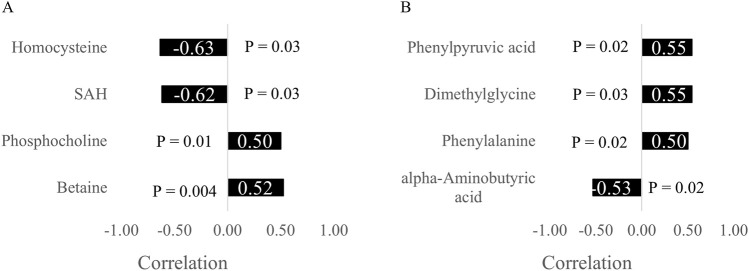
Correlations between rectal temperature, the Pectoralis major muscle **(A)** and liver **(B)** metabolites.

Very few metabolites were significantly correlated with chick rectal temperature. In the breast muscle (Figure 7A), only four metabolites exhibited a significant correlation with the rectal temperature. These metabolites included: homocysteine, S-adenosyl-homocysteine (SAH), phosphocholine, and betaine. Homocysteine and SAH exhibited negative correlations while phosphocholine and betaine exhibited positive correlations with rectal temperature.

In the liver, four different metabolites were also found to be significantly correlated with rectal temperature (Figure 7B). These metabolites included: phenyl pyruvic acid, dimethylglycine, phenylalanine, and alpha-aminobutyric acid. Only this last metabolite exhibited a negative correlation while all other three metabolites exhibited positive correlations with rectal temperature.

## 4 Discussion

In our previous study, we demonstrated that *in ovo*-fed amino acids, specifically methionine, cysteine, and leucine were associated with lower facial temperatures as well as lower oxidative damage, as measured by lipid peroxidation products in the serum and in the liver, under heat stress conditions. We argued that these changes reflected an improved tolerance to higher ambient temperatures in finishing broiler chickens (Yehia et al., 2024). The current study is a pilot study that aims to explore the changes in amino acids metabolism-related pathways that were influenced by the *in ovo*-fed amino acids in the muscle and liver tissue using targeted metabolomics at 24 h post-hatch, prior to the application of the heat stress program. The reasoning underlying the choice of 24 h post-hatch for sampling is that if these *in ovo*-fed amino acids induce metabolic changes during the late phase of embryogenesis by a yet to be determined mechanism, which in turn contribute to the improved heat tolerance later in life, these changes will be more evident early post-hatch. Muscle and liver were selected for this study due to their importance for thermoregulation.

### 4.1 Pathway analysis

In this pilot study, we used a multivariate approach and specifically, a sparse partial least square–discriminant analysis (sPLS-DA) coupled with a cross-validation scheme with many repeats to identify the subset of metabolites that were contributing to and maximize the separation between the experimental groups. We then used these model-selected metabolites to conduct the pathway enrichment analysis.

In both muscle and liver samples taken from treated chicks, metabolites retained by the model (Supplementary Tables S1, S2) were significantly enriched in pathways mainly related to the antioxidant defense system (Tables 2, 4), which was not the case for the CTRL group (Tables 1, 3). The enrichment of the cysteine and methionine metabolism pathway in the muscle of the *in ovo*-injected group is in coherence with the experimental treatments as both T1 and T2 included methionine and cysteine. This pathway is tightly associated with the glutathione metabolism pathway (Anderson, 1998), which was also significantly enriched in the muscle and liver of the *in ovo*-injected group. In the methionine cycle, methionine is converted to S-adenosyl-methionine (SAM). SAM is then demethylated to S-adenosyl-homocysteine (SAH), which is then hydrolysed into homocysteine. Homocysteine enters the trans-sulphuration pathway to form cysteine, the rate-limiting amino acid for the synthesis of glutathione (Zhang et al., 2022). Glutathione (GSH) is the substrate of antioxidant enzymes such as glutathione peroxidase. GSH is also a scavenger of free radicals, and an integral component of the antioxidant defense system (Lugata et al., 2024).

The glycine, serine, and threonine metabolism pathway was also significantly enriched in muscle and liver samples from chicks that were *in ovo*-fed with experimental treatments (Tables 2, 4). It has been shown that supplementing broiler diets with 2.0% of glycine was associated with a significant increase in serum total antioxidant capacity (T-AOC) and in the activity of glutathione peroxidase (GSH-PX) under heat stress conditions, reflecting an improvement in resistance to heat stress (Deng et al., 2023). Glycine is synthesized from serine and threonine, and it is used for the biosynthesis of glutathione, which explains its importance for the antioxidant defense system (Wang et al., 2013).

Glutamate is another constituent of glutathione (Wang et al., 1998). It can be synthesized from proline (Wu et al., 2011), which also has been shown to have a free radical scavenging activity *in vitro* (Kaul et al., 2008). Consequently, the significant enrichment of the proline metabolism pathway in muscle and liver tissue of treated chicks potentially indicate an indirect contribution of this pathway to the antioxidant defense system.

In the present study, the taurine metabolism pathway was found to be significantly enriched in muscle and liver samples from both treated groups (Tables 2, 4). Taurine, a sulfur-containing non-proteinogenic β-amino acid, has been shown to exert a protective effect under chronic heat stress conditions by reducing protein degradation in the pectoral muscles, facilitating lipolysis for energy and enhancing protein synthesis (Lu et al., 2019). As a result, taurine partially alleviates the impact of heat stress on broiler performance and carcass quality (Lu et al., 2019). Moreover, dietary taurine supplementation is also associated with a significant and linear increase in the activity of antioxidant enzymes as well as a significant decrease in malondialdehyde in the liver and serum of broiler chickens that were fed with increasing concentrations between 0.0 and 7.5 g/kg of dietary taurine (Han et al., 2020). Taurine exerts its antioxidant effects by stabilizing the mitochondria, where free radicals are produced, as its deficiency is known to compromise the mitochondrial electron transport chain, resulting in a significant increase in free radical production and subsequent oxidative damage (Surai et al., 2021). Taurine is thus vital for the antioxidant defense system. Additionally, taurine supplementation was reported to decrease triglycerides concentration in the liver and in the serum, which further reduce the potential for lipid peroxidation and oxidative damage (Han et al., 2020).

The histidine metabolism pathway was found to be significantly enriched in both the muscle and liver tissues of the treated groups (Tables 2, 4). L-Histidine has many antioxidant properties such as the capacity to scavenge hydroxyl radicals and singlet oxygen (Wade and Tucker, 1998). Kopec et al. (2016) fed female turkeys with a diet supplemented with 0.18% of L-Histidine for 103 days and found increased radical scavenging capacity in both the plasma and breast muscles. Additionally, L-Histidine is a major component of histidine-containing dipeptides (Boldyrev et al., 2013). Lackner et al. (2021) fed broilers with diets containing increased ratios of histidine/lysine between 0.44 and 0.64. These authors reported a significant increase in plasma carnosine concentrations, which grew proportionally with the increasing histidine/lysine ratio. Carnosine is well known for its antioxidant properties. For instance, it has been shown that the pre-treatment of muscle satellite cells with carnosine before the exposure to oxidative stress was associated with a significant reduction in intracellular reactive oxygen species (ROS) and in protein carbonyls, indicating reduced oxidative damage (Palin et al., 2020). Histidine and its metabolites are thus an integral part of the antioxidant defense system.

The arginine biosynthesis pathway was also found to be significantly enriched in muscle and liver of treated chicks (Tables 2, 4). Arginine can be catabolized into agmatine, a precursor of polyamines such as putrescine, spermine and spermidine, which have been shown to exert antioxidant effects (Fathima et al., 2024). Duan et al. (2015) supplemented the diet of broiler breeders with increasing levels if arginine from 0.96% to 1.36%. These authors reported that as the digestible arginine level increased in this range, the T-AOC of the egg yolk, liver, and breast muscles of the hatched chicks significantly increased. In addition, these authors reported a significant increase in the activity of the antioxidant enzyme GSH-PX in the serum, liver, and breast muscles of the day-old chicks. In the study of Duan et al. (2015), the increase in T-AOC and GSH-PX was associated with a significant decrease in the lipid peroxidation products, reflecting an improved antioxidant status. Lu et al. (2022) *in ovo*-fed 5.0 mg of arginine/egg and evaluated the GSH and malondialdehyde concentrations in breast muscles of hatched chicks on the day of hatch. These authors found a significant increase in GSH coupled with a significant decrease in malondialdehyde, demonstrating thus that arginine increased antioxidant substrates and reduced secondary lipid peroxidation products in muscle tissue. Furthermore, arginine can also be catabolized into ornithine and subsequently into L-Glutamate and proline (Fathima et al., 2024), which also exert antioxidant effects and contribute to the antioxidant defense system as previously discussed.

In muscle tissue of the *in ovo*-fed chicks in our study, the branched-chain amino acid biosynthesis pathway was not significantly enriched. This was expected as this pathway is downregulated by the presence of leucine (Salmon Kirsty et al., 2006). However, this pathway was significantly enriched in the liver tissue, which was not expected.

Overall, the data from this current study shows that *in ovo* feeding of methionine, cysteine and leucine activates pathways heavily involved in the response of the antioxidant defense system, which is in line with data from previous studies in which these three amino acids were *in ovo* fed and resulted in enhanced antioxidant status under heat stress or after embryonic thermal manipulation (Elnesr et al., 2019; Elwan et al., 2019; Ajayi et al., 2022; Han et al., 2022).

### 4.2 Rectal temperature-correlated metabolites

SAH is a methionine cycle metabolite that is further catabolized into homocysteine, another metabolite implicated in the same cycle (Parkhitko et al., 2019). Both metabolites were negatively correlated with chick rectal temperature in the present study. The accumulation of homocysteine has multiple harmful consequences for the cell including oxidative stress, mitochondrial dysfunction, and apoptosis (Shakeri and Le, 2022). Betaine, which was found to be positively correlated with the rectal temperature in the current study, serves as a methyl donor in the reaction catabolized by the enzyme betaine-homocysteine methyltransferase and converting homocysteine into methionine (Ueland et al., 2005). According to these correlations, as rectal temperature increases, betaine concentration also increases. Consequently, more betaine is available to donate methyl to the conversion of homocysteine to methionine, reducing thus the toxic effects of homocysteine including oxidative stress.

Phosphocholine, which was found to be positively correlated with rectal temperature of the sampled chicks of our study, is an intermediary metabolite in the *do novo* biosynthesis pathway of phosphatidylcholine (Gibellini and Smith, 2010). The concentration of certain phosphatidylcholines including 18:1/20:3, 18:0/18:1 and 18:1/18:1 was shown to increase in the liver under the effect of heat stress to help the liver adapt by stabilizing cell membranes, adjusting energy metabolism, and protecting hepatic cells against further damage (Guo et al., 2021).

Phenyl pyruvic acid is a product of the transamination of the alanine side chain of phenylalanine by the enzyme phenylalanine transaminase (Schuck et al., 2015). Both phenyl pyruvic acid and phenylalanine were found to be positively correlated with chicks’ rectal temperature in the present study. Very little is known about the role of these metabolites in thermoregulation. In a study on embryonic thermal manipulations (38.6°C for 10 h/d from ED 10 – ED 18), the concentration of phenylalanine was found to significantly decrease in the liver of chicks exposed to thermal manipulation compared to chicks from the control group at ED 14, but the concentration of this amino acid was very similar between the two groups at the day of hatch, suggesting a potential role of this amino acid in the response to heat stress (Han et al., 2017).

Dimethylglycine is formed in the mitochondria of the liver during the re-methylation of homocysteine to methionine, a reaction in which a methyl group is removed from betaine (i.e., trimethylglycine) converting it to dimethylglycine (Chalvatzi et al., 2020). In broiler diets, a supplementation of 1.0 g of dimethylglycine/kg of feed significantly reduced blood plasma malondialdehyde levels by 56% compared to the control group indicating an antioxidant effect of dimethylglycine (Kalmar et al., 2011). Dimethylglycine has also been shown to alleviate heat stress-induced intestinal damage by limiting the impact of heat stress on villus heights and goblet cell count (Wang et al., 2022).

Overall, very few to no data is available in the literature to help in interpreting the biological significance of these metabolites for thermoregulation and rectal temperature. Further research is thus needed to explore and explain the relationship between the identified metabolites and the rectal temperature in broiler chickens under variable environmental conditions.

## 5 Conclusion

This study aimed to explore the mechanisms underlying the effects of *in ovo*-fed amino acids on broiler antioxidant defense system and physiological temperature via the characterization of the amino acid metabolism in the breast muscle and liver during the early post-hatch period. The findings indicated that *in ovo* feeding of L-Methionine, L-Cysteine and L-Leucine did not alter hatchability or body weight of hatched chicks, which are traits of economic importance for the poultry industry.

The metabolomic data showed that *in ovo* feeding of sulfur (methionine and cysteine) and branched chain (leucine) amino acids led to the activation of multiple important pathways implicated in the response to oxidative stress in the muscle and the liver. Specifically, *in ovo* feeding of these amino acids activated the methionine, glutathione, and histidine metabolism pathways. It could thus be argued that the reduced oxidative damage in the liver and serum that we reported in our previous study in finishing broilers that were *in ovo*-fed with these amino acids (Yehia et al., 2024) could have been the result of these amino acids affording chicks an enhanced antioxidant response. A future study will be conducted to confirm this hypothesis.

Regarding the relationship between these *in ovo* fed amino acids and body temperature, several of the metabolites that were significantly correlated with rectal temperature also have multiple biological functions including antioxidant activity. Given that oxidative stress is one of the main deleterious consequences of heat stress, it could be speculated that by reducing the oxidative damage in the muscle and the liver, these metabolites could indirectly influence the overall response of broilers to heat stress, including their ability to thermoregulate and resist heat stress. Further research is required to understand the specific role of these metabolites in thermoregulation under thermoneutral and heat stress conditions.

## Data Availability

The original contributions presented in the study are included in the article/Supplementary Material, further inquiries can be directed to the corresponding author.
